# Acquisition of Gonococcal AniA-NorB Pathway by the Neisseria meningitidis Urethritis Clade Confers Denitrifying and Microaerobic Respiration Advantages for Urogenital Adaptation

**DOI:** 10.1128/iai.00079-23

**Published:** 2023-04-24

**Authors:** Yih-Ling Tzeng, Soma Sannigrahi, Zachary Berman, Emily Bourne, Jennifer L. Edwards, Jose A. Bazan, Abigail Norris Turner, James W. B. Moir, David S. Stephens

**Affiliations:** a Division of Infectious Diseases, Department of Medicine, Emory University School of Medicine, Atlanta, Georgia, USA; b Department of Pediatrics, The Research Institute at Nationwide Children’s Hospital and The Ohio State University, Columbus, Ohio, USA; c Division of Infectious Diseases, Department of Internal Medicine, The Ohio State University College of Medicine, Columbus, Ohio, USA; d Sexual Health Clinic, Columbus Public Health, Columbus, Ohio, USA; e Department of Biology, University of York, Heslington, York, United Kingdom; f Department of Microbiology and Immunology, Emory University School of Medicine, Atlanta, Georgia, USA; Universite de Geneve

**Keywords:** NO, *Neisseria gonorrhoeae*, *Neisseria meningitidis*, denitrification, urethritis

## Abstract

Neisseria meningitidis historically has been an infrequent and sporadic cause of urethritis and other urogenital infections. However, a nonencapsulated meningococcal clade belonging to the hyperinvasive clonal complex 11.2 lineage has recently emerged and caused clusters of urethritis cases in the United States and other countries. One of the genetic signatures of the emerging N. meningitidis urethritis clade (*Nm*UC) is a chromosomal gene conversion event resulting in the acquisition of the Neisseria gonorrhoeae denitrification apparatus—the N. gonorrhoeae alleles encoding the nitrite reductase AniA, the nitric oxide (NO) reductase NorB, and the intergenic promoter region. The biological importance of the N. gonorrhoeae AniA-NorB for adaptation of the *Nm*UC to a new environmental niche is investigated herein. We found that oxygen consumption, nitrite utilization, and NO production were significantly altered by the conversion event, resulting in different denitrifying aerobic and microaerobic growth of the clade. Further, transcription of *aniA* and *norB* in *Nm*UC isolates differed from canonical N. meningitidis, and important polymorphisms within the intergenic region, which influenced *aniA* promoter activity of the *Nm*UC, were identified. The contributions of three known meningococcal regulators (NsrR, FNR, and NarQP) in controlling the denitrification pathway and endogenous NO metabolism were distinct. Overall, transcription of *aniA* was dampened relative to canonical N. meningitidis, and this correlated with the lower NO accumulation in the clade. Denitrification and microaerobic respiration were bolstered, and protection against host-derived NO was likely enhanced. The acquisition of the N. gonorrhoeae denitrification pathway by the *Nm*UC supports the clade’s adaptation and survival in a microaerobic urogenital environment.

## INTRODUCTION

Neisseria meningitidis remains a worldwide cause of bacterial meningitis in children and young adults and rapidly fatal sepsis in otherwise healthy individuals (1, 2). N. meningitidis is an obligate human pathogen carried predominantly in the nasopharynx by up to 3 to 10% of adults in nonepidemic periods (3), and carriage can be significantly higher (>30%) in some populations (4, 5). Historically, N. meningitidis has been reported as a sporadic cause of urethritis and other urogenital infections but with very low overall prevalence (6–9). However, since 2015, urethritis cases in predominantly heterosexual men were found to be caused by the United States N. meningitidis urethritis clade (US_NmUC) (hereafter referred to as *Nm*UC or the “clade”) (10, 11), a capsule-deficient clade belonging to the hyperinvasive clonal complex (cc) 11.2 lineage (12–14). As an example, a significant portion (≈20%) of epidemiologically unlinked, presumed Neisseria gonorrhoeae urethral infections in Columbus, Ohio from 2015 to 2016 were determined to be caused by *Nm*UC with clinical presentation mirroring that of gonococcal urethritis (13–15). *Nm*UC is unique in causing urethritis outbreaks (16) and case clusters, which have been identified in 14 states in the United States (17), the United Kingdom (18, 19), and Vietnam (20). Other mucosal infections (neonatal conjunctivitis) (21) and cases of invasive disease caused by clade isolates also have been identified (17, 22).

**FIG 1:**
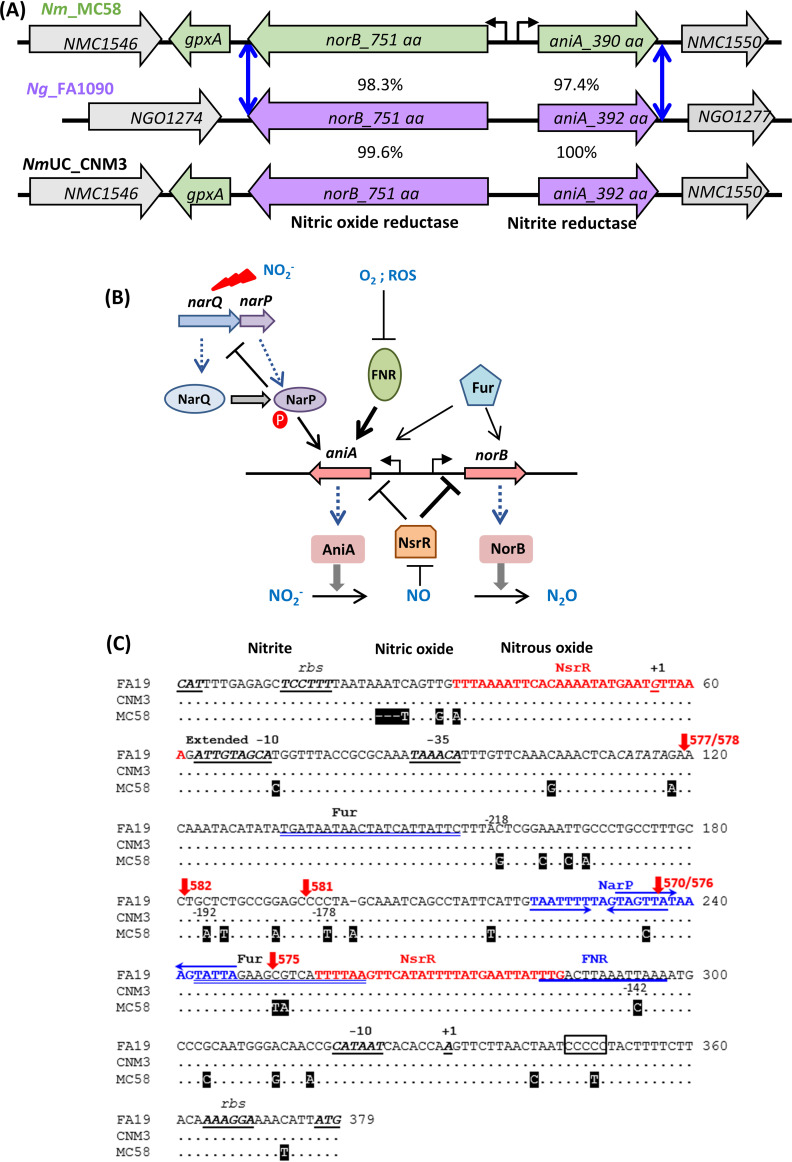
(A) Schematics of the *norB-aniA* locus in the canonical N. meningitidis strain MC58, N. gonorrhoeae strain FA1090, and N. meningitidis urethritis clade (*Nm*UC) isolate CNM3. Blue double arrowhead lines indicate the recombination junctions of the gene conversion event in the clade (10). Protein sequence similarities (%) between MC58 and FA1090 and between FA1090 and CNM3 are shown for the corresponding *norB* and *aniA* genes. (B) Regulatory network of the *aniA-norB* locus. Regulation of the denitrification pathway by NarQP (36, 38), FNR (35–37), Fur (40, 41), and NsrR (38, 39) has been described in N. meningitidis. (C) Alignment of the *norB-aniA* intergenic sequences of gonococcal strain FA19, *Nm*UC isolate CNM3, and meningococcal strain MC58. Polymorphisms differing from the FA19 sequence are shown. The promoter elements (−10, extended −10 and −35), ribosomal binding site (rbs), and transcriptional start site (+1) are underlined and italicized. The FNR motif is marked with a blue line, the NsrR motifs are shown in red, the Fur motifs are double underlined in blue, and the NarP binding site with two sets of inverted repeats is labeled in blue. The SNP locations relative to the *aniA* start codon as described in hybrid promoter studies are labeled beneath the sequence. The N. gonorrhoeae reference strains, FA19 and FA1090, have 5 and 6 Cs in a short poly-C track, respectively (boxed). The junctions of the N. meningitidis-N. gonorrhoeae hybrid promoter are indicated with red vertical arrows together with each construct’s name as in Fig. 8.

The meningococcus can evolve quickly and adapt as a result of frequent horizontal genetic exchanges via transformation. All clade isolates (>200 geographically and temporally dispersed isolates) have acquired, via a precise chromosomal gene conversion event, the genetically distinct N. gonorrhoeae denitrification apparatus. This (gonococcal) apparatus is comprised of the nitrite reductase AniA, the nitric oxide (NO) reductase NorB, and the gonococcal regulatory intergenic region (IGR) that separates the divergent transcripts (10) (Fig. 1A). This genetic feature is not shared by other reported sporadic N. meningitidis urethritis isolates (23–25). AniA catalyzes the conversion of nitrite to NO that is subsequently reduced to nitrous oxide by NorB. These two proteins enable the utilization of nitrite as an alternative respiratory electron acceptor (26), and gonococci universally have highly functional AniA and NorB. In contrast, a functional AniA is not essential for meningococci (27, 28), and many meningococcal isolates have various mutations in *aniA* or completely lack the *aniA* gene (26–28). AniA is a glycosylated, surface-exposed outer membrane lipoprotein (28). It is the major gonococcal protein induced anaerobically (29), is expressed during gonococcal infection (30), and is shown to confer enhanced serum resistance to gonococci (29). Thus, AniA is being investigated as a gonococcal vaccine antigen (31–33). There is also evidence that biofilm formation may occur during natural gonococcal infection, and *aniA* and *norB* are two highly upregulated genes in biofilms (34). NorB additionally plays a significant role in protection against toxicity elicited by NO, which is formed endogenously via bacterial respiration and produced by epithelial and phagocytic host cells, both of which are encountered during infection. Hence, the AniA-NorB denitrification pathway plays a crucial role in gonococcal biology and pathogenesis during urogenital infection.

Several transcriptional regulators, including the fumarate and nitrate regulator (FNR) (35–37), NsrR (38, 39), Fur (40, 41), and the nitrate/nitrite anaerobic respiration, NarQ/NarP, two-component system (TCS) (36, 38), control *aniA* and/or *norB* expression in N. meningitidis and/or N. gonorrhoeae (Fig. 1B). Expression of *aniA* is (i) induced by FNR in response to oxygen limitation (35–37), (ii) induced by nitrite via the NarQ/P TCS (36, 38), (iii) activated by the iron uptake regulator Fur (41), and (iv) repressed by the NO-responsive repressor NsrR (38, 39, 42). The clade’s IGR represented by the CNM3 sequence (Fig. 1C), which is found in 97% of 255 *Nm*UC isolates, is identical to that of N. gonorrhoeae and distinct from the meningococcal IGR (10) and likely contributes to regulatory changes in the *Nm*UC. As AniA and NorB are involved in respiration using nitrite and/or nitric oxide as alternative electron acceptors, we define the changes in metabolic pathways, microaerobic/anaerobic growth, and regulatory phenotypes afforded by the gonococcal *aniA/norB* conversion. These alterations most likely contributed to the emergence of *Nm*UC as an urethrotropic pathogen.

## RESULTS

### Transition from aerobic to microaerobic denitrification growth.

Acquisition of the genetically distinct N. gonorrhoeae denitrification apparatus is found in 97% of 255 sequenced *Nm*UC isolates (17, 43) currently in the PubMLST database. Protein sequence alignments of AniA and NorB of N. meningitidis MC58 and N. gonorrhoeae FA1090 showed 97.4% and 98.3% similarity, respectively (Fig. 1A). AniA from a representative *Nm*UC isolate, CNM3, was 100% identical to that of FA1090, whereas the CNM3 NorB had three amino acid residues that differed from that of N. gonorrhoeae FA1090 (99.6% similarity). Compared to another N. gonorrhoeae reference strain FA19, there are two amino acid residues and a single residue change in AniA and NorB, respectively. For comparison, there are 12 and 13 residue differences between AniA and NorB of N. meningitidis MC58 and N. gonorrhoeae FA1090, respectively.

**FIG 2:**
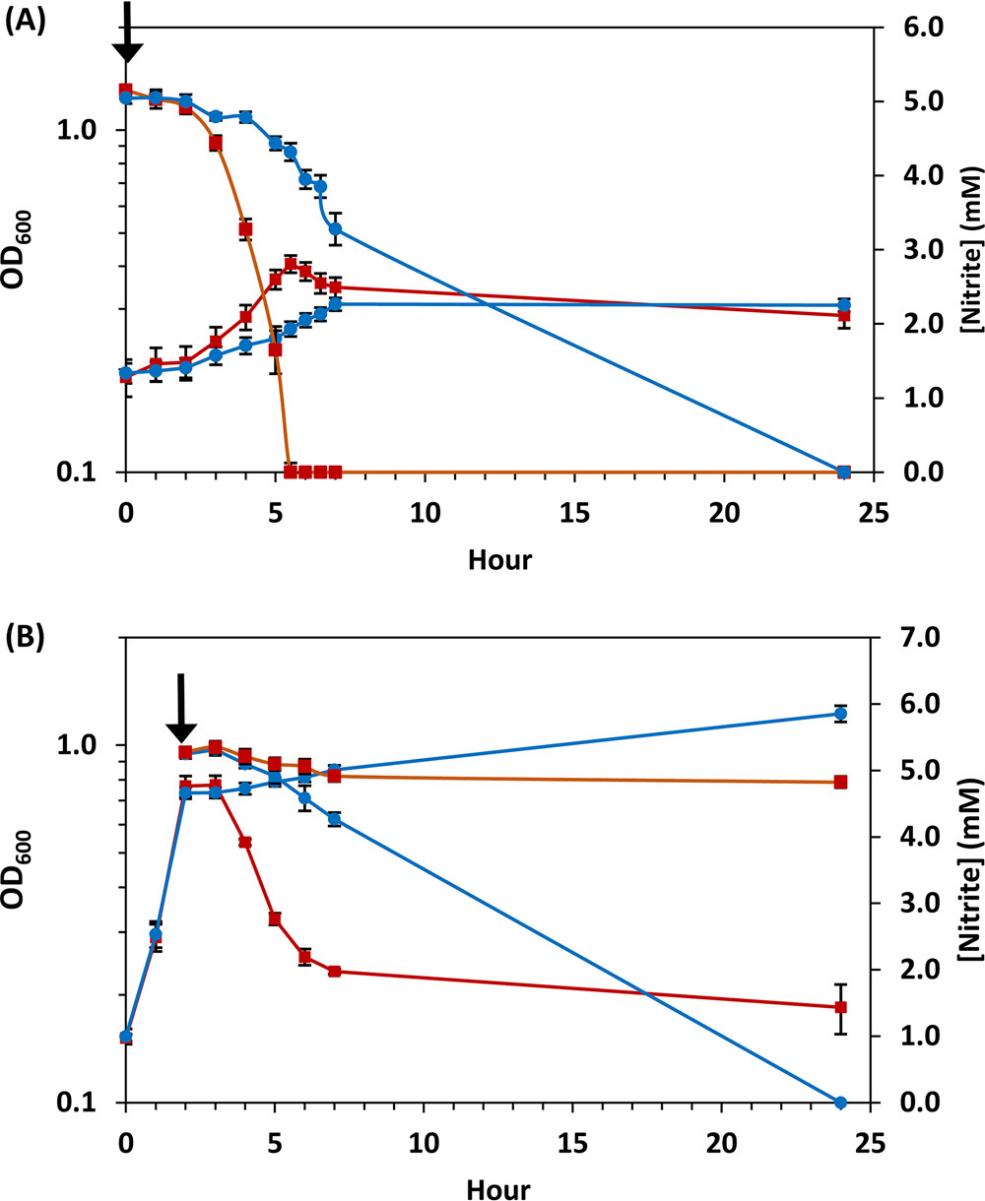
(A) Growth and nitrite concentrations of microaerobic cultures of MC58 (■, red) and CNM3 (•, blue) in supplemented GC broth with 5 mM nitrite (arrow) added at inoculation. The OD_600_ values (left axis) and nitrite concentrations (right axis in mM) over 24 h are plotted. Data shown are geometric means and standard errors of the mean of four independent experiments performed in duplicates. (B) Growth and nitrite concentrations of aerobic GC broth cultures with 5 mM nitrite added at logarithmic phase (arrow) representing a “nitrite shock” scenario are plotted as in panel A. The geometric means and standard errors of the mean of three independent experiments done in duplicates are shown.

Growth of CNM3 and the reference N. meningitidis invasive strain, MC58, was compared under three physiologically relevant growth conditions using different respiratory electron acceptors as follows: aerobic and microaerobic with or without nitrite. Strain MC58 has maintained a functional meningococcal denitrification system and is the most extensively characterized meningococcal strain for nitrite-dependent growth (27, 41, 44, 45). MC58 routinely grew aerobically to a higher optical density than CNM3. Under microaerobic conditions, growth of both strains was substantially impaired. The addition of nitrite improved growth, suggesting that nitrite acts as an alternative electron acceptor. When nitrite concentrations were measured during microaerobic growth (Fig. 2A), MC58 consumed nitrite rapidly and reached maximal culture density when nitrite was depleted. In contrast, CNM3 used nitrite at a lower rate and thus corresponding slower growth. Growth was also examined when rapidly switching from aerobic oxygen respiration to denitrifying respiration (38). When nitrite was added to actively growing aerobic cultures (a nitrite shock scenario) (38), the growth of MC58 was quickly halted with minimal nitrite consumption, and the culture density subsequently decreased, indicating cell lysis (Fig. 2B). In contrast, CNM3 immediately started to reduce nitrite and exhibited sustained growth. Thereby, unlike meningococci with N. meningitidis AniA-NorB (e.g., MC58), CNM3 could more readily switch from oxygen to nitrite as the respiratory electron acceptor.

**FIG 3:**
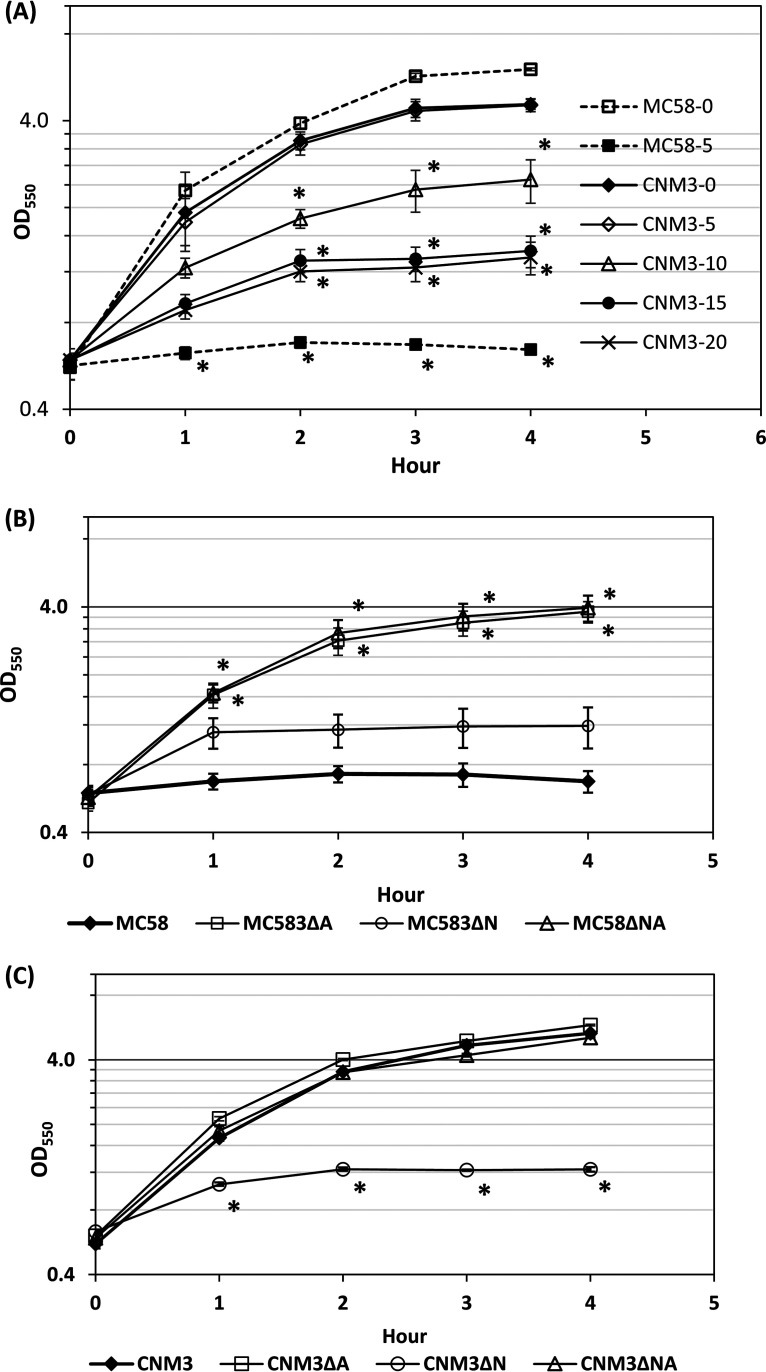
(A) Growth in the presence of increasing nitrite concentrations in which nitrite was added to log-phase aerobic GC broth cultures (nitrite shock condition). CNM3 cultures (solid line) with 0 (♦), 5 mM (◊), 10 mM (Δ), 15 mM (•), and 20 mM (×) nitrite as well as MC58 cultures (dash lines) with 0 (□) and 5 mM (■) nitrite were examined (*n* = 4). The time point when nitrite was added into cultures was set as T = 0. (B) Aerobic GC broth cultures of MC58 in the presence of 5 mM nitrite are shown for WT (◊), ΔA (□), ΔN (ο), and ΔNA (Δ) mutants (*n* = 5). (C) Aerobic GC broth cultures of CNM3 in the presence of 5 mM nitrite are shown for WT (◊), ΔA (□), ΔN (ο), and ΔNA (Δ) mutants (*n* = 3). The geometric means and standard errors of the mean are plotted. Two-tailed unpaired Student’s *t* tests were performed to compare with cultures without nitrite. *, *P* < 0.01.

The effects of nitrite concentrations were next examined using actively growing aerobic cultures that were exposed to nitrite. Growth of CNM3 was not affected by the presence of 5 mM nitrite, tolerated 10 mM nitrite, and continued to grow, albeit at a decreased rate, in the presence of 20 mM nitrite (Fig. 3A). In contrast, the addition of 5 mM nitrite completely inhibited MC58 growth. To confirm the role of the AniA-NorB pathway in the observed growth differences, single (*aniA* [ΔA] and *norB* [ΔN]) and double (*aniA* and *norB* [ΔNA]) mutants were created in both CNM3 and MC58, and growth in the presence of 5 mM nitrite was compared. As shown in Fig. 3B, the MC58ΔA and MC58ΔNA mutants grew well, whereas the wild type (WT) and the MC58ΔN mutant grew poorly. The restored growth of MC58ΔA suggested that NorB activity in WT MC58 was unable to handle the amount of endogenous NO produced by AniA. In contrast, only the CNM3ΔN mutant showed impaired growth with 5 mM nitrite (Fig. 3C). The additional mutation in *aniA* rescued the growth defect of the CNM3ΔN mutant (i.e., CNM3ΔNA mutant). The ΔNA mutants of both MC58 and CNM3 grew normally in the presence of nitrite, indicating that the growth inhibition was due to NO, not nitrite *per se*. Taken together, these data pointed to probable NO toxicity that affected meningococcal denitrifying growth and that was a detrimental consequence of higher than optimal AniA and/or insufficient NorB activities in canonical meningococci.

### Differences in oxygen consumption and NO production in N. meningitidis during denitrifying microaerobic growth.

**FIG 4:**
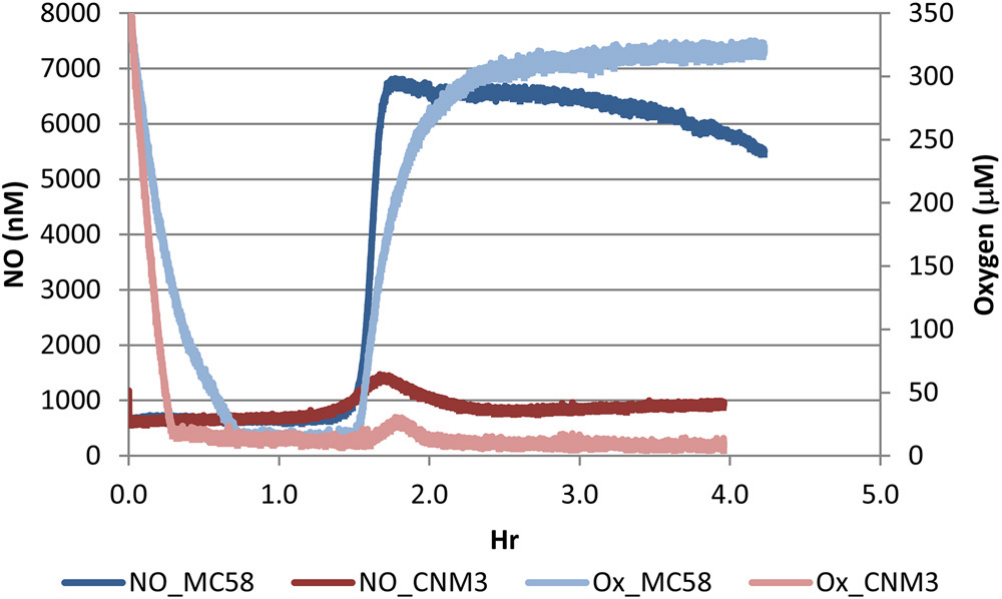
Measurement of nitric oxide accumulation and oxygen change over time. GC broth cultures supplemented with 5 mM nitrite were stirred in a Clark electrode chamber with continuous recording in milliseconds. Oxygen tensions of CNM3 (pink) and MC58 (light blue) initially decline over time but increase again when denitrification has started. Subsequent accumulation of NO during transition to denitrification is different between CNM3 (red) and MC58 (blue). A representative of three independent experiments of CNM3 and MC58 is shown here, while the complete data are included in Fig. S1 in the supplemental material.

The different rates of nitrite utilization between CNM3 and MC58 (Fig. 2) suggested changes in endogenous NO levels. To assess this possibility, we measured the NO concentration and oxygen tension simultaneously in cultures supplemented with 5 mM nitrite and grown in Clark electrode chambers (46). Differences between CNM3 and MC58 were observed in the rates of oxygen consumption, the subsequent accumulation of NO during transition to denitrification, as well as the recovery of oxygen tension. A representative data set plotting [NO] and [O_2_] in CNM3 and MC58 cultures of three measurements is shown in Fig. 4 (see complete data in Fig. S1 in the supplemental material). At the onset of each measurement, oxygen tension dropped rapidly for both strains. After aerobic respiration depleted oxygen, both cultures switched to nitrite-dependent respiration. MC58 produced NO much faster and to a higher level (blue), whereas NO accumulated slowly and at a significantly lower peak level for CNM3 (red), at most reaching one-third of MC58’s peak level (Fig. S1). The NO concentration in the CNM3 culture then declined, in contrast to the sustained high NO level in MC58 over the course of the experiment (Fig. 4; see also Fig. S1). There were statistically significant differences between the two strains by two-tailed unpaired Student's *t* test (see Table S3 in the supplemental material), both in the maximal NO concentrations reached during the time course measurements (*P = *0.006) and in the sustained levels at the 3-h time points (*P = *0.007). Once MC58 initiated NO production, oxygen again started to accumulate and remained at a saturated level (light blue). However, in the CNM3 cultures, oxygen only slightly increased in concert with changes in NO levels (pink, Fig. 4). At 3-h, there were statistically significant differences in oxygen tensions between MC58 and CNM3 (*P = *0.034). This dissimilarity in oxygen accumulation was presumably due to differential NO inhibition of the respiratory oxygen-reducing enzyme, cytochrome oxidase, which is known to be inhibited by NO (47). Overall, these profiles reflected modified microaerobic oxygen and nitrite respiration dynamics, i.e., electron flow, in *Nm*UC, in which the gonococcal AniA-NorB apparatus replaced that of meningococci.

### Transcription of *aniA* and *norB* in the *Nm*UC differed from canonical meningococci.

**FIG 5:**
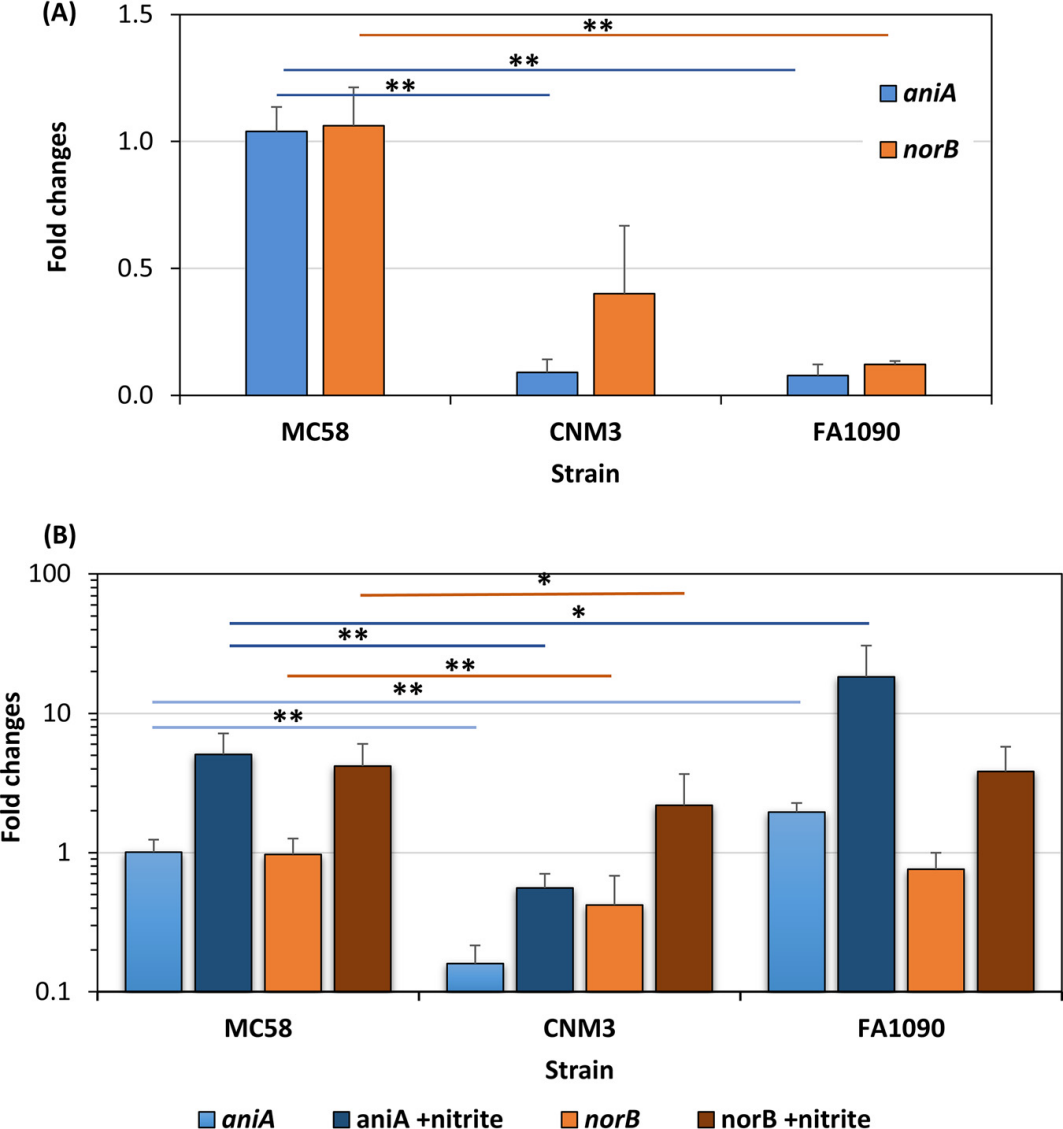
(A) Basal expression of *aniA* and *norB* in aerobic cultures of MC58, CNM3, and FA1090 without nitrite as determined by qRT-PCR. Fold change in gene expression is normalized to *aniA* (blue) and *norB* (orange) of MC58 cultures without nitrite (*n* ≥ 4). (B) Expression in microaerobic cultures of MC58, CNM3, and FA1090 with or without 5 mM nitrite (*n* ≥ 5). Fold changes in gene expression in the presence of 5 mM nitrite are shown with darker colors. The mean values and standard deviations are shown. Two-tailed unpaired Student’s *t* tests were performed to compare two groups, and those with statistically significant differences are indicated by lines. *, *P* < 0.05; **, *P* < 0.01.

The data above demonstrated clear phenotypic differences between CNM3 and MC58 in microaerobic and denitrification growth mediated by the AniA-NorB pathway, supporting a biological consequence of the N. gonorrhoeae gene conversion event. Thus, a detailed study of gene expression and regulation was warranted to understand the changes in *aniA-norB* expression compared to the typical N. meningitidis and N. gonorrhoeae that have differential regulations and expression profiles (38, 41). The NO level is a balance between AniA-mediated production and NorB-controlled NO metabolism. We tested the hypothesis that the drop in MC58 viability during nitrite shock conditions was associated with higher *aniA* expression in MC58 relative to CNM3 in the absence of nitrite, which could lead to excess NO production upon nitrite addition. RNAs were prepared from aerobic cultures without nitrite for reverse transcription-quantitative PCR (qRT-PCR). When normalized to expression in MC58 (Fig. 5A), *aniA* in CNM3 was expressed at ≈11-fold lower levels, whereas no significant difference was detected between MC58 and CNM3 for *norB* expression. Another N. meningitidis urethritis clade isolate, CNM10, yielded expression patterns similar to CNM3 (*aniA*, 0.14 ± 0.06, *P* < 0.001; *norB*, 1.02 ± 0.75) compared to those in MC58. For comparison, N. gonorrhoeae FA1090 expressed significantly lower *aniA* and *norB* relative to MC58 during aerobic growth (Fig. 5A). These results indicated that the MC58 *aniA* was not tightly suppressed during aerobic growth when compared to *aniA* in gonococci and *Nm*UC, both of which encode the gonococcal denitrification genes. The “leaky” production of *aniA* in MC58 together with similar *norB* expression results in an excess of endogenous NO upon sudden nitrite exposure, causing NO toxicity and cell lysis. As both N. meningitidis and N. gonorrhoeae can simultaneously cometabolize oxygen and nitrite as electron acceptors, we also tested the situation by adding nitrite to aerobic cultures at inoculation. When RNAs were extracted from cultures exposed to nitrite at inoculation to steadily induce the denitrification pathway, more *norB* was expressed than *aniA* in all three strains (*aniA/norB* ratios of 0.14 ± 0.07, 0.43 ± 0.06, and 0.25 ± 0.12 for CNM3, MC58, and FA1090, respectively). Accordingly, the steady induction of both *aniA* and *norB* allowed manageable NO levels with utilization of both oxygen and nitrite as electron acceptors (i.e., growth in Fig. 2A).

RNAs from microaerobic cultures of MC58, CNM3, and FA1090 in the absence and presence of nitrite were also examined. Induced gene expression of *fnr*, which encodes the major regulator for adaptation to oxygen-limiting conditions (36), was indeed observed by qRT-PCR when compared to those of aerobic cultures (data not shown), confirming the microaerobic nature of the experimental condition. In the absence of nitrite, *aniA* of CNM3 was expressed approximately 5- and 10-fold lower than those of MC58 and FA1090, respectively (Fig. 5B). The nitrite-induced *aniA* expression was ≈5-, 3-, and 9-fold in MC58, CNM3, and FA1090, respectively, with CNM3 demonstrating the lowest level. In contrast, the expression of *norB* occurred at similar levels among the three strains, with or without nitrite. Overall, under microaerobic conditions, the most notable transcriptional change in the clade, resulting from the gonococcal gene conversion, was the considerably dampened *aniA* expression. Assuming analogous AniA and NorB enzymatic activities between N. meningitidis and N. gonorrhoeae, these data correlated with the lower NO accumulation in the CNM3 cultures (Fig. 4).

### Intergenic promoter sequences influenced expression of *aniA* and *norB*.

Expression of *aniA* and *norB* is controlled by divergent promoters within the intergenic region (IGR). The gene conversion event also resulted in the exchange of the meningococcal IGR to a gonococcal sequence (Fig. 1C). To better define the impact of a gonococcal IGR on the expression of *aniA* and *norB* in the clade, translational reporters (48), in which expression of the *lac* reporter gene is dependent on an in-frame fusion to an open reading frame with a promoter and a ribosomal binding site (*rbs*) for translational initiation, were used to further assess the overall transcriptional and post-transcriptional regulation. The reporters were integrated into the chromosome as a single copy (49) in strain CNM3. The *aniA*::*lacZ* and *norB*::*lacZ* reporters contained IGR sequences from MC58 (C552 and C555, respectively, for the representative meningococcus) or from CNM3 (C554 and C557, respectively, for the clade and the gonococcus). The profiles of C552 (*aniA_Nm_*) and C555 (*norB_Nm_*) would, thus, represent typical meningococci because CNM3 otherwise carries meningococcal regulators. Conversely, because the clade harbors meningococcal regulators, but gonococcal promoters within the IGR, a hybrid mechanism of *aniA* (C554) and *norB* (C557) regulation would be expected to occur. Activities of both the N. meningitidis and N. gonorrhoeae promoters were compared in the same CNM3 background, thereby standardizing the contributions of regulators and enable a better evaluation of the influences of promoter sequence variations. In addition, all of these reporters were also successfully generated in the gonococcal strain FA19, where the *aniA_Ng_* and *norB_Ng_* reporters (F554 and F557, respectively) represented the typical gonococcal expression and the *aniA_Nm_* and *norB_Nm_* reporters (F552 and F555, respectively) represented how meningococcal promoters may interact with the gonococcal regulatory network. There was a single cytosine difference in a short poly-C track within IGR (boxed in Fig. 1C) between FA19 and FA1090, which is used as the N. gonorrhoeae reference in qRT-PCR analysis (Fig. 5). Using the 378-bp CNM3 IGR sequence, which is 100% identical to FA19, to query the 15,796 N. gonorrhoeae genome collections that have *aniA* tagged in PubMLST (in January 2023), this sequence was found in 8,248 genomes (52.2%), and 7,030 N. gonorrhoeae genomes (44.5%) have the IGR of FA1090. Thus, the FA19 IGR sequence shown in Fig. 1C represents a broad collection of N. gonorrhoeae strains.

**FIG 6:**
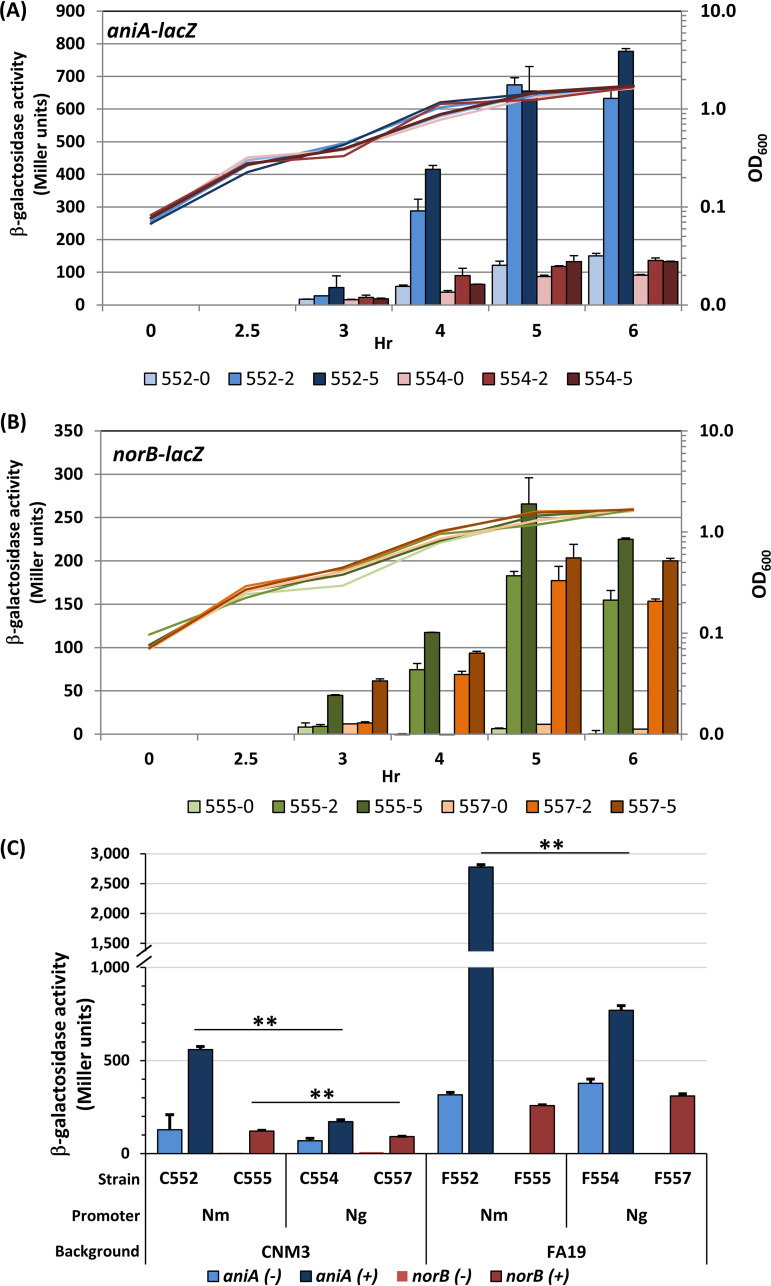
(A) Growth phase-dependent β-galactosidase activities of the CNM3 *aniA*::*lacZ* translational reporter. Strains C552 (*aniA_Nm_*, blue) and C554 (*aniA_Ng_*, red) were monitored during aerobic growth with nitrite concentrations of 0 (light blue and light red bars), 2 mM (blue and red bars), or 5 mM (dark blue and dark red bars). Growth curves measured at every hour are colored as the corresponding bar graphs and shown as lines without data points on a semilogarithmic scale of the secondar*y* axis. All growth curves are very similar to each other, and there are no significant differences under different nitrite concentrations. (B) Growth phase-dependent β-galactosidase activities of the CNM3 *norB*::*lacZ* translational reporters (C555, *norB_Nm_* and C557, *norB_Ng_*) as shown in panel A. (C) β-galactosidase activities of cultures grown under microaerobic conditions for 20 h in the presence (+) or absence (−) of 5 mM nitrite (*n* = 3). The *aniA* reporters in CNM3 are C552 and C554 and, in the FA19 background, are F552 and F554, respectively. C555 and C557 are the *norB* reporters in CNM3, whereas F555 and F557 are in FA19. Two-tailed unpaired Student’s *t* tests were performed to compare N. meningitidis and N. gonorrhoeae promoter in the same genetic background. Lines with double asterisks indicate statistically significant difference (**, *P* < 0.01). All reporters are significantly more active in FA19 than in CNM3 by two-tailed unpaired Student’s *t* tests (*P* < 0.01) with the exception of the *norB* reporters (C555, F555, C557, and F557) under the no nitrite condition, which yielded minimal units.

The dose-dependent effect of nitrite on reporter activity was examined under aerobic conditions and with nitrite added at inoculation. Reporter activity was highest for all strains during the early stationary phase (Fig. 6A and B). Without nitrite, the *aniA_Nm_* promoter (light blue) was slightly more active than the *aniA_Ng_* promoter (light pink). When nitrite was added, the *aniA_Nm_* promoter (C552) was induced significantly (*P* < 0.001) and reached much higher activities (2 mM, blue; 5 mM, dark blue) than the *aniA_Ng_* promoter sequence (C554), which was minimally induced (red and dark red) (Fig. 6A). In contrast, the nitrite inductions of both the *norB_Nm_* and *norB_Ng_* promoters were significant (*P* < 0.001, except 2 mM nitrite at 3 h postinoculation) and reached comparable levels for the N. meningitidis and N. gonorrhoeae/clade promoters (Fig. 6B). Under the same nitrite levels, C552 (*aniA_Nm_*) yielded much higher Miller units (MU) than those of C555 (*norB_Nm_*) (blue in Fig. 6A versus green in Fig. 6B), whereas comparable activities were observed between C554 (*aniA_Ng_*) and C557 (*norB_Ng_*) (red and orange in Fig. 6A and 6B, respectively). The differences again indicated that *aniA* expression mediated by the N. meningitidis promoter was much higher than that derived from the gonococcal promoter in a meningococcal background. As the *aniA_Nm_* and *aniA_Ng_* promoter comparisons were in the same genetic background, the transcriptional differences were mainly due to intergenic sequence variations.

The responses of reporters to nitrite under microaerobic conditions were next examined in the CNM3 and FA19 backgrounds (Fig. 6C). In the absence of nitrite (light blue), similar activities were found between *aniA_Nm_* and *aniA_Ng_* promoters, and minimal *norB* promoter activities were observed in both genetic backgrounds. Both *aniA* promoters were more active in FA19 than in CNM3. When exposed to nitrite, the *aniA_Nm_* promoter was much more active than the *aniA_Ng_* promoter regardless of the strain background. In contrast, the *norB_Nm_* and *norB_Ng_* promoters were induced to similar levels in both CNM3 and FA19. The gonococcal *aniA_Ng_* and *norB_Ng_* promoters in a matched gonococcal FA19 background were more active than the *aniA_Nm_* and *norB_Nm_* promoters in a matched meningococcal CNM3 background. The mismatched N. gonorrhoeae promoter sequence with the N. meningitidis regulatory network resulted in markedly diminished expression of *aniA_Ng_* but yielded only a slight change in *norB_Ng_* expression (the clade’s situation). Curiously, the meningococcal promoters were much more active when cloned into the gonococcus. In particular, the induction of *aniA_Nm_* was considerably higher than when *aniA_Nm_* was controlled by meningococcal regulators (2,776 ± 40 and 558 ± 17 MU for F552 and C552, respectively), implying that the N. gonorrhoeae and N. meningitidis regulatory networks acted differently toward a noncognate promoter sequence.

**FIG 7:**
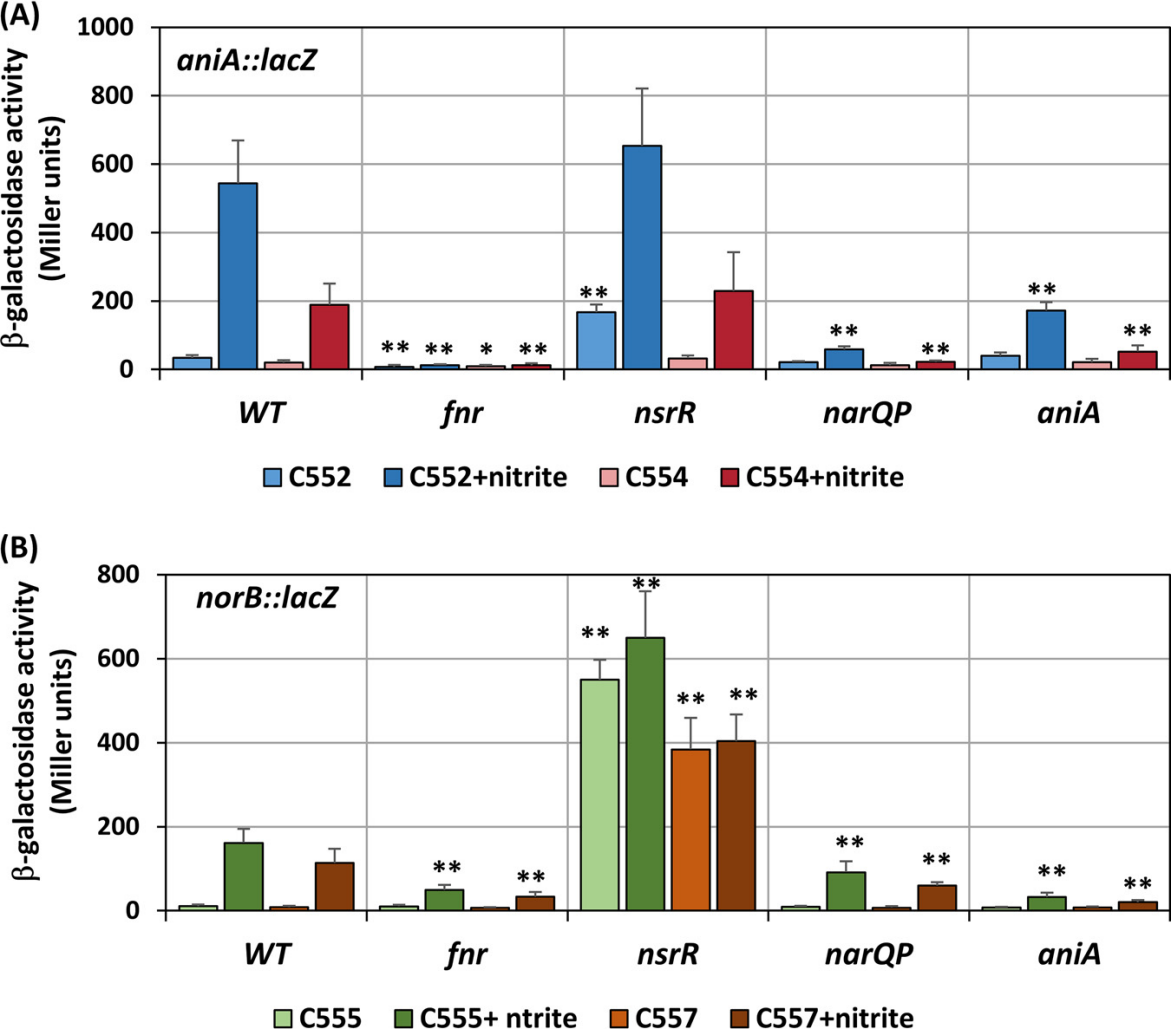
Activities of the *aniA*::*lacZ* (A) and *norB*::*lacZ* (B) reporters in the CNM3 wild type and the *fnr*, *nsrR*, *narP*, and *aniA* backgrounds. Samples were collected from 20-h microaerobic cultures with or without 5 mM nitrite (*n* ≥ 3). Two-tailed unpaired Student’s *t* tests were performed for comparison between the wild type and mutants under the same conditions. *, *P* < 0.05; **, *P* < 0.01.

Finally, as NO is a critical signal controlling the denitrification pathway, the effects of endogenous NO were further examined by deleting *aniA* in the reporter strains outlined above. In the CNM3ΔA background, nitrite-induced *aniA_Nm_* and *aniA_Ng_* activities decreased 3.2- and 3.6-fold, respectively (*P* ≤ 0.01) when compared to those of the parental WT strains (Fig. 7). Endogenous NO is not synthesized in an *aniA* mutant to derepress NsrR. Thus, the residual increase in the *aniA* reporter activity in response to nitrite was likely due to the nitrite-sensing NarQP TCS. Decreases of 5.0- and 5.3-fold in the CNM3ΔA background relative to the WT strains were also seen for *norB_Nm_* and *norB_Ng_*, respectively (*P *≤ 0.01). Induction of *norB* also occurred in the presence of exogenously added NO donor, *S*-nitroso-l-glutathione (50) (data not shown). Taken together, these data again confirmed the involvement of NO in the regulation of both denitrification enzymes.

### Contributions of meningococcal regulators to *aniA* and *norB* expression in *Nm*UC.

Expression of *aniA* is regulated by FNR in response to anaerobic conditions (35–37) as well as by the NarQ-NarP TCS (36, 38). AniA-mediated reduction of nitrite to NO also relieves NsrR, a NO-responsive repressor (38, 39, 42), and augments *aniA* expression. Thereby, using the CNM3 reporter strains, we generated mutations in these known regulators to assess the regulation of *aniA* and *norB* under microaerobic and microaerobic denitrifying conditions (Fig. 7).

### (i) Mutation in *fnr*.

In the absence of nitrite, the *fnr* mutation caused a further reduction in *aniA_Nm_*_,_ (34 ± 8 versus 7 ± 6 MU; *P* < 0.01) and *aniA_Ng_* (20 ± 6 versus 9 ± 4 MU; *P* < 0.05) expression. The addition of 5 mM nitrite was unable to induce the expression of *ani*A in the *fnr* mutant, confirming that FNR is essential for activating *aniA* transcription. Conversely, both *norB_Nm_* and *norB_Ng_* promoters remained inducible by nitrite, but the *fnr* mutation caused an approximately 3-fold decrease in expression when compared to that of the WT.

### (ii) Mutation in *nsrR*.

Two NsrR binding motifs have been identified within the *norB* and *aniA* IGRs, and they are identical between N. meningitidis and N. gonorrhoeae (Fig. 1C). Thus, we hypothesized that the outcomes of an *nsrR* mutation would likely be similar between the N. meningitidis and the N. gonorrhoeae/clade promoters if not affecting other regulators. This was confirmed for *norB* expression—maximal levels of derepression were observed in the *nsrR* mutants for both *norB_Nm_* and *norB_Ng_* reporters (C555 and C557, respectively), as nitrite did not further enhance *norB* expression (Fig. 7). This suggests that NsrR is the dominant (negative) regulator of *norB* expression in N. gonorrhoeae/clade as well as in N. meningitidis.

Dissimilar changes between *aniA_Nm_* and *aniA_Ng_* reporters were observed in the *nsrR* mutant. In the absence of nitrite, transcription of the *aniA_Nm_* in the *nsrR* mutant was 5-fold higher than that of WT (167 ± 23 versus 34 ± 8 MU; *P* < 0.01), but the expression of *aniA_Ng_* showed no significant change (32 ± 9 versus 20 ± 6 MU, *P = *0.1). Whereas the *nsrR* mutation resulted in maximal *norB* expression regardless of the presence of nitrite, *aniA* expression remained inducible by nitrite, with *aniA_Nm_* induced approximately 3-fold higher than *aniA_Ng_* (Fig. 7A). The difference in nitrite-induced *aniA_Nm_*_,_ and *aniA_Ng_* levels in the *nsrR* mutant implied that one or more regulators yielded differential activation strengths on *aniA_Nm_* and *aniA_Ng_* promoters in the absence of NsrR.

### (iii) Mutation in *narP*.

The NarQ (kinase) and NarP (response regulator) TCS responds to nitrite and induces *aniA* expression in N. meningitidis (45). Two overlapping NarP-binding sites were mapped upstream of the NsrR and FNR binding motifs, which were near the *aniA* promoter element but distal to the *norB* promoter (38). As such, we anticipated no direct NarP-mediated regulation of *norB* and, consistently, transcription of *norB* in the *narP* mutant was not significantly different from the WT (*P* ≥ 0.1). The ≈40% reduction in *norB* expression induced in the *narP* mutants relative to WT were likely an indirect effect caused by decreased *aniA* expression and, thus, lower NO levels. However, without NarQP, nitrite induction of *aniA* was only ≈10% of WT levels for both *aniA* reporters, pointing to an important role for the nitrite-sensing NarQP TCS in the upregulation of denitrification. Although considerably lower than that detected in the WT, *aniA* expression remained inducible (≈2-fold) by nitrite in the *narP* mutants.

### Mapping important polymorphisms influencing the *aniA* expression.

**FIG 8:**
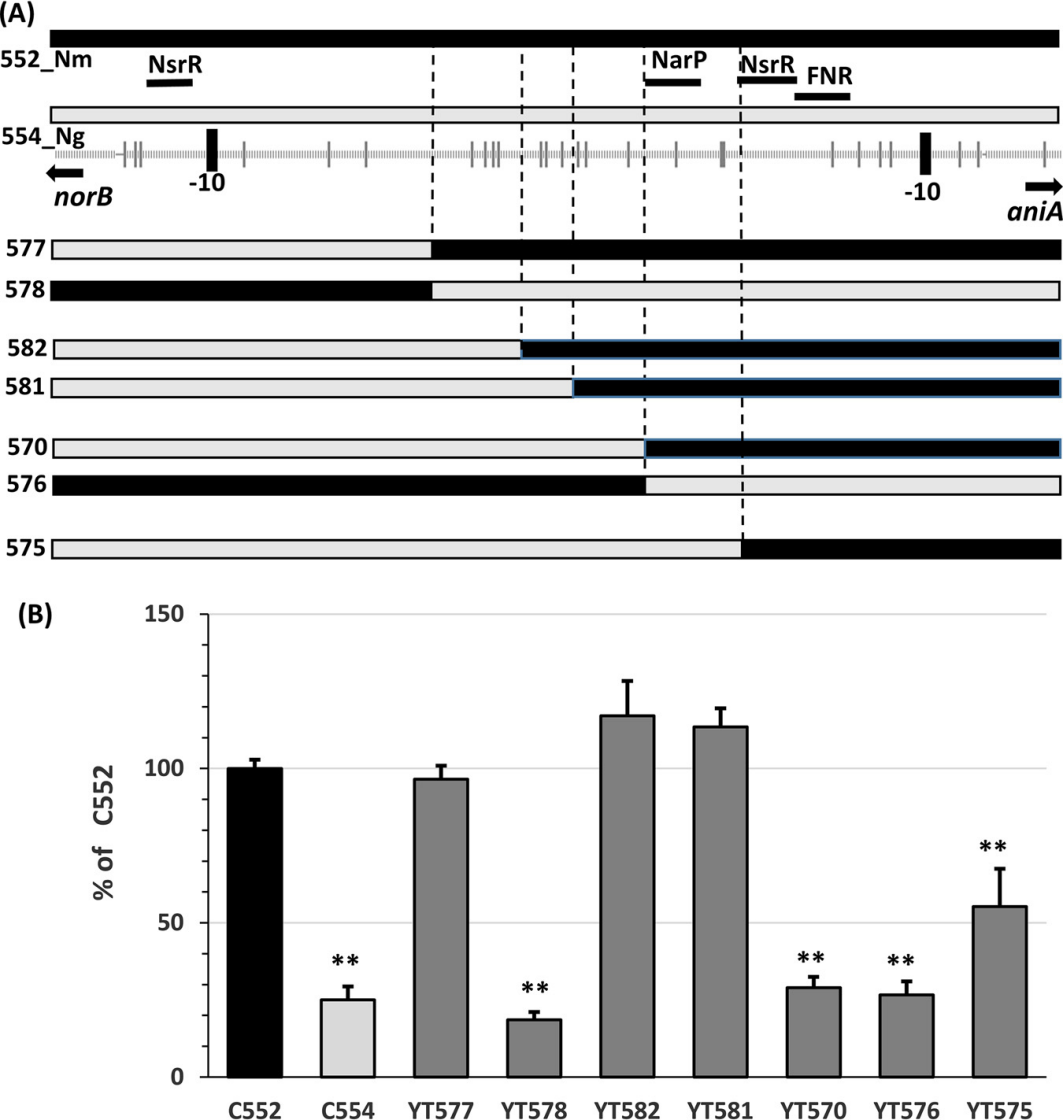
(A) A Schematic of N. meningitidis-N. gonorrhoeae hybrid *aniA*::*lacZ* promoter constructs. The meningococcal MC58 sequences are indicated as black lines and the N. gonorrhoeae FA1090 sequences as gray lines. The locations of SNPs are marked as vertical lines below, whereas the locations of binding motifs for NsrR, NarP, and FNR are labeled as black bars above the N. gonorrhoeae sequence. (B) β-Galactosidase activities of the *aniA*::*lacZ* reporters in CNM3 with the wild-type N. meningitidis (C552), the wild-type N. gonorrhoeae (C554), or hybrid sequences between N. meningitidis and N. gonorrhoeae promoters as in panel A. Samples were collected from overnight (20 h) microaerobic cultures with 5 mM nitrite. Data for each promoter construct were recorded as Miller Units and normalized to Miller Units recorded for the wild-type N. meningitidis reporter (C552), set as 100% (*n* ≥ 4). Two-tailed unpaired Student’s *t* tests were performed for comparison between C552 and other fusion promoter constructs. **, *P* < 0.01.

The N. gonorrhoeae and clade IGRs are distinct from meningococcal IGRs with 28 single nucleotide polymorphisms (SNPs) (Fig. 1C) that likely contributed to the changes in the transcription of *aniA* and *norB*. To better define the *cis* element critical to the regulation of the gonococcal denitrification pathway, now acquired by the *Nm*UC, we examined the effects of sequence divergence using hybrid promoters of *aniA_Nm_* and *aniA_Ng_*. We replaced the upstream sequences in the *aniA_Nm_* reporter with the N. gonorrhoeae sequence (Fig. 8A) to allow a systematic identification of SNPs that affected the *aniA_Nm_* promoter. When needed, the converse hybrid promoter constructs were made. First, fusion promoters (YT577 and YT578) (Fig. 8A) containing all SNPs up to the C/G change at −218 bp upstream of the *aniA* start codon recapitulated the activities of N. meningitidis and N. gonorrhoeae reporters with the entire IGR (Fig. 8B). Two reporters, YT582 and YT581, which included SNPs up to either −192 (C/A) or −178 (C/T), respectively, also preserved activities analogous to that of the entire *aniA_Nm_* promoter (C552). However, the fusion reporter YT570 encompassing the −142 (T/C) SNP, located within the proposed NarP binding motif, yielded lower MUs equivalent to the *aniA_Ng_* reporter (C554). Three SNPs are found between YT581 and YT570 (two T→C changes and a deletion of A) (Fig. 1C). Thus, the lower transcription in YT570 relative to YT581 suggested that these three SNPs were a part of the NarP binding motif necessary for full NarP-mediated activation. The converse fusion YT576 has the three N. meningitidis SNPs but with the N. gonorrhoeae SNP in the NarP-binding motif. YT576 yielded a reporter activity like that of YT570, suggesting that these four N. meningitidis SNPs together result in higher *aniA* expression in YT581 relative to the sequences with mismatched N. meningitidis and N. gonorrhoeae SNPs (YT570 and YT576) and to the N. gonorrhoeae sequence (YT578). Finally, the YT575 reporter yielded a higher *aniA* transcription relative to YT578. Since both reporters carried the same N. gonorrhoeae NarP-binding motif, the higher YT575 reporter activity likely resulted from the N. meningitidis consensus FNR-binding motif in YT575, whereas YT578 has the N. gonorrhoeae FNR motif with an SNP that weakens interactions with FNR (41).

## DISCUSSION

Branched electron-transfer networks enable bacteria to use diverse electron acceptors for respiration. *Neisseria* spp. possess AniA and NorB, which can use nitrite and/or NO as alternative respiratory substrates during microaerobic growth (26, 41, 51). Although denitrification is previously considered as an anaerobic process, both N. meningitidis and N. gonorrhoeae can cometabolize oxygen and nitrite as electron acceptors during oxygen-limited growth (41, 45, 52). Interestingly, many meningococcal strains have various mutations in *aniA* (26–28). Moir has proposed that the meningococcus is on an evolutionary trajectory toward a loss of the capacity to reduce nitrite and is evolving to become a NO-tolerant aerobe in an oxygen-rich human nasopharyngeal niche (53). Gonococcus, on the other hand, is a facultative anaerobe that uses nitrite when oxygen is limited. A unique genomic signature of the recently emerged *Nm*UC is the acquisition of gonococcal AniA-NorB via a complete and precise gene conversion event (10). Historically, the rare, sporadic N. meningitidis urogenital isolates are often nonfunctional in denitrification (10, 19, 23), and none have acquired the N. gonorrhoeae denitrification apparatus reported in *Nm*UC. As shown by our data, acquisition of gonococcal NorB-AniA and the resulting changes to *aniA* and *norB* expression create a unique capacity of the *Nm*UC to modify the amount of endogenous NO, influence respiratory electron flow, and minimize NO toxicity. These beneficial changes potentially affect the fitness of the *Nm*UC, thus facilitating the transition of colonization of the oxygen-rich nasopharynx to the urogenital niche, which is not a strictly anaerobic but rather an oxygen-limited environment (54). Our studies have limitations. To compare to earlier reports, we adopted experimental conditions previously used for characterization of gonococcal and meningococcal denitrification pathways (36, 38, 39, 44), which are not likely to be encountered *in vivo*. The “nitrite shock with 5 mM nitrite” scenario reported by Overton et al. (38) has been used for studying NO homeostasis in gonococci and the ability of switching respiratory electron acceptors when suddenly exposed to high concentrations of nitrite in mediating gonococcal growth. While growing bacterial cultures at atmospheric oxygen level is a standard practice and is used in this study as aerobic culture conditions, oxygen levels in ambient air of *in vitro* cultures and the nitrite concentrations examined here are higher than the physiological levels encountered *in vivo* (55). For example, oxygen tension at oral/nasal sites is 83 to 145 mm Hg, the urinary tract is 0.5 to 52 mm Hg, and the vagina is 15 to 35 mm Hg (54). The oxygen tension at cervix ranges between 12 and 22 mm Hg (50).

Our data demonstrated that altered nitrite metabolism occurred between a representative *Nm*UC isolate, CNM3, and a canonical N. meningitidis, MC58. Anjum et al. (44) found that nitrite impairs aerobic growth but enhances microaerobic growth of MC58. We confirmed these prior observations for MC58 but also showed that aerobic growth of CNM3 was not significantly impaired by nitrite at concentrations of up to 10 mM. After initiating nitrite utilization in MC58, faster and higher NO production sustained a blockade of oxygen-dependent respiration. This most likely was the result of terminal cytochrome oxidase inhibition, leading to an exclusive use of nitrite. In contrast, NO concentration in CNM3 remained low (Fig. 4). The growth defects observed for the *norB* mutants of both MC58 and CNM3 were rescued by concurrent inactivation of *aniA* (Fig. 3), suggesting that excess NO, not nitrite, was toxic.

The data demonstrated clear phenotypic differences between CNM3 and MC58 in microaerobic and denitrification growth mediated by the AniA-NorB pathway, supporting a biological consequence of N. gonorrhoeae gene conversion event. We then proceeded to a detailed study of gene expression and regulation that included understanding how the N. gonorrhoeae IGR of *Nm*UC changes *aniA* and *norB* expression compared to that of the typical N. meningitidis and N. gonorrhoeae. The expression of both meningococcal and gonococcal AniA and NorB proteins is under the control of a complex regulatory network (26). The *Nm*UC contains hybrid regulatory elements—gonococcal intergenic promoters and meningococcal regulatory proteins. Therefore, we anticipated that expression of the denitrification pathway in the *Nm*UC would be different from canonical meningococci (41). We observed that *aniA* in MC58 was not tightly suppressed during aerobic growth (Fig. 5A). This “leaky” production of AniA in MC58 yielded excess NO upon sudden nitrite exposure, resulting in NO toxicity and cell death. The significantly dampened *aniA* expression in CNM3 relative to MC58 allowed for manageable NO accumulation with the utilization of both nitrite and oxygen as electron acceptors (Fig. 5A). Under microaerobic conditions, CNM3 again demonstrated significantly dampened transcription of *aniA* relative to MC58 (Fig. 5B). Although the *in vitro* enzymatic activity of N. gonorrhoeae AniA is higher than that of N. meningitidis (27), our data showed that the disadvantage of acquiring a more active N. gonorrhoeae AniA in the *Nm*UC was compensated by lowering *aniA* expression. There is no biochemical comparison of NorB activities between N. meningitidis and N. gonorrhoeae, and we showed that *norB* expression in *Nm*UC was only decreased modestly in comparison to MC58. Hence, the reduction of *aniA* expression could be a major contributor to the success of *Nm*UC in adapting to the microaerobic urethra.

We performed reporter studies in the same CNM3 genetic background; therefore, the mismatch between N. meningitidis regulators and the N. gonorrhoeae promoter sequence provides one explanation for the clade’s unique *aniA* and *norB* expression profiles. Under both aerobic and microaerobic conditions, the basal levels of *aniA* and *norB* in the absence of nitrite were similar between N. meningitidis and N. gonorrhoeae, but nitrite induced significantly higher *aniA_Nm_* expression relative to *aniA_Ng_* (Fig. 6). Under microaerobic conditions, nitrite-induced expression of both the *aniA_Nm_* and *norB_Nm_* reporters increased 16-fold, whereas the *aniA_Ng_* and *norB_Ng_* reporters increased 10-fold (Fig. 7). This implies that the meningococcal regulators interact less optimally with the gonococcal promoter. A reduction in AniA also would result in a reduction in NO available for NO-mediated derepression of *aniA*, leading to a further overall decrease in *Nm*UC compared to N. meningitidis. Whereas NarPQ are responsive to nitrite in N. meningitidis, NarPQ regulation of the N. gonorrhoeae denitrification pathway occurs independently of nitrite (38). Thereby, in the *aniA* mutant, the modest increase in *aniA_Nm_* reporter activity, which was not observed for *aniA_Ng_*, most likely can be attributed to the nitrite-sensing activity of N. meningitidis NarPQ (38). Using hybrid *aniA* promoter constructs, we determined that three SNPs upstream of the originally mapped NarP motif were essential for higher transcription originating from the *aniA_Nm_* promoter relative to the *aniA_Ng_* promoter.

The *aniA_Nm_* and *aniA_Ng_* reporters in CNM3 responded differently to an *nsrR* mutation from those observed in gonococci, wherein *aniA* expression in the *nsrR* mutant did not respond to nitrite and was at a level comparable to nitrite-induced expression in the WT (38). In the absence of nitrite, the *aniA_Nm_* expression was 5-fold higher in the *nsrR* mutant than in the WT. In contrast, *aniA_Ng_* activity was only slightly higher than the WT upon inactivation of NsrR. There are no differences between the *aniA_Nm_* and *aniA_Ng_* NsrR binding sequences; thus, the differential response to an *nsrR* mutation likely can be attributed to other regulators. In contrast, the effect of an *nsrR* mutation on *norB* expression was consistent with NsrR being a dominant repressor, in which both *norB_Nm_* and *norB_Ng_* activities reached maximal derepressed levels that were independent of NO, parallel to that observed in gonococci. FNR is essential for any appreciable *aniA* transcription, as *aniA* was minimally expressed in the *fnr* mutant, even when nitrite was present to initiate NarP induction and NsrR derepression. A biochemical binding study shows that N. meningitidis FNR interacts weakly with the N. gonorrhoeae *fnr* motif carrying one SNP difference from the N. meningitidis motif (41) (Fig. 1C). This divergence from N. meningitidis, a fully conserved *fnr* consensus sequence also found in Escherichia coli and other Gram-negative bacteria (37), may in part account for the low *aniA_Ng_* expression (41). No FNR-binding site is near the *norB* promoter. Thus, the markedly decreased *norB* expression in the *fnr* mutants was likely caused by the minimal *aniA* expression, which would result in decreased endogenous NO to counter the repression by NsrR. Taken together, the “quieter” expression of *aniA* in *Nm*UC requires both the faulty FNR box and the 3 SNPs (2× T→C and a deletion of “A”) just upstream of the NarP-binding motif.

The *Nm*UC has been predominantly isolated from the male urethra and not from the female genital tract. Whereas cervical gonorrhea is often asymptomatic, infection of the male urethra typically results in an acute inflammatory response including the release of NO (47, 56). A study by Overton et al. (38) has proposed that host-derived NO induction of the N. gonorrhoeae NorB could confer a survival advantage *in vivo* by serving as an electron acceptor under conditions of oxygen limitation within the urogenital tract. *Neisseria* spp. respond to oxygen limitation by inducing alternative denitrification respiration. By significantly dampening AniA induction, while retaining a high level of NorB production, the *Nm*UC maintains endogenous NO accumulation at a low level and can lessen the impact of NO produced by host inflammatory responses during acute urethritis. Thus, the gonococcal AniA-NorB system promotes *Nm*UC survival during the transition to the microaerobic environment of the urogenital tract by bolstering denitrifying and microaerobic respiration and likely by conferring protection against host-derived NO.

## MATERIALS AND METHODS

### Bacterial isolates and growth conditions.

Bacterial strains used in this study are listed in Table S1 in the supplemental material. CNM3 served as the major representative of the *Nm*UC (10). *Neisseria* isolates were cultured on GC base agar containing 0.4% glucose and 0.68 mM Fe(NO_3_)_3_ at 37°C and 5% CO_2_ or in GC broth with the same supplements and 0.043% NaHCO_3_ as the CO_2_ source at 37°C. Brain heart infusion medium with 1.25% fetal bovine serum was used with kanamycin selection. Antibiotic concentrations (μg/mL) used for *Neisseria* (E. coli) were as follows: kanamycin, 80 (50); chloramphenicol, 5 (34); tetracycline, 5; and spectinomycin, 60 (100). All growth experiments were conducted with standard supplemented GC broth. A 12-mL culture in a 15-mL tube at 100 rpm aeration that reached maximal *fnr* induction after overnight incubation was defined as the condition for microaerobic growth, whereas 10 mL in a 50-mL tube at 200 rpm was defined as the aerobic condition. Denitrifying cultures contained 5 mM nitrite unless noted differently.

### Measurements of nitrite and NO.

Nitrite was assayed colorimetrically with the Griess reagent as previously described (44). Reaction mixtures contained 5 μL of cell suspension and 895 μL of a 1% sulfanilamide solution, and the reactions were then started by the addition of 100 μL of a 0.02% solution of *N*-naphthylenediamine. Absorbance was recorded at optical density at 540 nm (OD_540_). Oxygen tensions and NO concentrations were continuously monitored with a Clark-type oxygen electrode (Rank Brothers) and with an iso-NO electrode (World Precision Instruments) (46).

### Construction of mutants.

All primers used for the genetic manipulation of bacteria are noted in Table S2 in the supplemental material.

### (i) Δ*aniA-norB*::*aphA3* (ΔNA) mutant.

A fragment downstream of *norB* (5n) was obtained by PCR using primers norB-3R and norB3FA3, and sequence downstream of *aniA* (3a) was amplified with aniA3FA3 and aniA-3R using CNM3 genomic DNA. The *ahpA3* cassette fragment (A3) was obtained using aphA3-SmF and aphA3-SmR. Mixtures of 5n and A3 were used as the template for the 1st round of overlapping PCR with primers gpxA-3R and A3-SmR. The resulting fragment was mixed with 3a and used for the second PCR with primers gpxA-3R and aniA-3R2 to obtain the final construct, in which the entire ≈4-kb *norB* and *aniA* coding sequence and the IGR had been deleted.

### (ii) Δ*aniA*::*aphA3* (ΔA) mutant.

The 5′ fragment of *aniA* was made using pnorB-lacR and aniA5RA3; while the 3′ fragment was obtained using aniA3FA3 and aniA-3R2. The final overlapping PCR with primers pnorBlacR and aniA-3R2 generated the ≈1.8-kb construct in which 1,317 bp of the *aniA* coding sequence were deleted.

### (iii) Δ*norB*::*aphA3* (ΔN) mutant.

The flanking fragments were obtained with primers gpxA-3R and norB3FA3 and primers norB5RA3 and paniA-lacR. The second overlapping PCR was performed with gpxA-3R and paniA-lacR to yield a ≈2.3-kb construct that deleted 2,031 bp of *norB*.

Genomic DNAs of *nsrR*::Ω (39), *fnr*::*erm* (36), and *narQP*::Ω (45) mutants were used as PCR templates. The *fnr*::*Erm* mutation was retrieved using primers fnr-5F1 and fnr-3R. Similarly, the *nsrR*::Ω(Sp) sequence with an internal 279-bp deletion was obtained using nsrR-5F2 and nsrR-3R. The PCR product of *narP*-*narQ* deletion was produced with narQ-5F1 and narP-3R. These PCR products were used to transform meningococci. The integration and mutation were confirmed by colony PCR with primers fnr-3R and fnr-5F2 (*fnr*), NsrR-3R and nsrR-5F1 (*nsrR*), and narQ-5F2 and narP-3R (*narPQ*).

### Reporter construction and β-galactosidase assay.

Promoter fragments with flanking BamHI sites were obtained by PCR using PnorB-lacR-Bm+PaniA-lacR-Bm for *aniA* reporters and aniA-lacR2+norB-lacR2 for *norB* reporters using Q5 polymerase (New England Biolabs) and cloned into pLES94 as translational fusions (49). The length of the promoter insert and the orientation were confirmed with proL2 and lacZrev and primers PnorB-lacR-Bm and lacZrev, respectively. The resulting plasmids were used to transform CNM3 and correct transformants identified by colony PCR. Regulator mutations were subsequently introduced into the appropriate reporters.

Site-specific changes in the fusion promoter studies were created by overlapping PCR primers at the desired locations and with the intended changes. Primer pairs used to generate promoter fusion plasmids were as follows: R-gc-216 and F-gc-216 for YT570, R-gc-264 and F-gc-264 for YT575, R-nm-214 and F-nm-214 for YT576 and pYT581, R-gm-143+F-gm-143 for YT577 and YT578, and R-202nm+F-202-nm for YT582. The presence of the desired nucleotide changes were verified by sequencing. β-Galactosidase activities were assayed in triplicate by the Miller method (57) and represented by Miller units, which are calculated as 1,000 × (OD_420_ − 1.75 × OD_550_)/(minute × mL × OD_600_).

### Quantitative RT-PCR.

Cultures collected at mid-log phase or after overnight microaerobic growth were treated with RNAprotect (Qiagen). Total RNA was isolated using RNeasy minikit (Qiagen), treated with Turbo DNase (Invitrogen), and purified with Quick RNA microprep kit (Zymo). The cDNA samples were obtained by reverse transcription of total RNA (1 to 0.5 μg) using GeneAmp RNA PCR core kit (Applied Biosystems), and reactions without the reverse transcriptase served as negative controls. The transcription of genes of interest was measured using the SYBR green detection method (Bio-Rad) (58). The internal control for normalization is 16s rRNA. Each qRT-PCR was performed in duplicate. A Student's *t* test with a two-tailed hypothesis was used to determine any significant difference (*P* < 0.01) between two variables.
