# Supplementary material for: Acquisition of Gonococcal AniA-NorB Pathway by the Neisseria meningitidis Urethritis Clade Confers Denitrifying and Microaerobic Respiration Advantages for Urogenital Adaptation
Source: Infect Immun. 2023 Apr 24;91(5):e00079-23. doi: 10.1128/iai.00079-23 (PMC10187123; doi:10.1128/iai.00079-23)
Supplement: Supplemental file 1 — Tables S1 to S3 and Fig. S1. Download iai.00079-23-s0001.pdf, PDF file, 0.7 MB [file iai.00079-23-s0001.pdf]

Supplemental Materials

Table S1. Strains used in this study

Name	Genotype
CNM3	*N. meningitidis, Nm*UC
CNM3ΔA	CNM3 with Δ*aniA::aphA3*
CNM3ΔN	CNM3 with Δ*norB::aphA3*
CNM3ΔNA	CNM3 with Δ*aniA-norB::aphA3*
CNM3, fnr	CNM3 with *fnr::Erm*
CNM3, nsrR	CNM3 with *nsrR::Ω(Sp)*
CNM3, narQP	CNM3 with *narQP:: Ω (Sp)*
MC58	*N. meningitidis*
MC58ΔA	MC58 with Δ*aniA::aphA3*
MC58ΔN	MC58with Δ*norB::aphA3*
MC58ΔNA	MC58with Δ*aniA-norB::aphA3*
FA19	*N. gonorrhoeae* reference
FA1090	*N. gonorrhoeae* reference
C552	CNM3 carrying the *aniA::lacZ* reporter of MC58 promoter
C554	CNM3 carrying the *aniA::lacZ* reporter of CNM3 promoter
C555	CNM3 carrying the *norB::lacZ* reporter of MC58 promoter
C557	CNM3 carrying the *norB::lacZ* reporter of CNM3 promoter
F552	*N. gonorrhoeae* FA19 carrying the *aniA::lacZ* reporter of MC58 promoter
F554	*N. gonorrhoeae* FA19 carrying the *aniA::lacZ* reporter of CNM3 promoter
F555	*N. gonorrhoeae* FA19 carrying the *norB::lacZ* reporter of MC58 promoter
F557	*N. gonorrhoeae* FA19 carrying the *norB::lacZ* reporter of CNM3 promoter
YT570	CNM3 carrying the *aniA::lacZ* reporter of pYT570 hybrid promoter
YT575	CNM3 carrying the *aniA::lacZ* reporter of pYT575 hybrid promoter
YT576	CNM3 carrying the *aniA::lacZ* reporter of pYT576 hybrid promoter
YT577	CNM3 carrying the *aniA::lacZ* reporter of pYT577 hybrid promoter
YT578	CNM3 carrying the *aniA::lacZ* reporter of pYT578 hybrid promoter
YT581	CNM3 carrying the *aniA::lacZ* reporter of pYT581 hybrid promoter
YT582	CNM3 carrying the *aniA::lacZ* reporter of pYT582 hybrid promoter

Table S2. Primers used in this study

Name	Sequence (5'-3')
norB-3R	CCCGGTGCGTTTACGATGGA
norB3FA3	ttcctcctagttagtcacccGGGACGGGAATGGAAAAGGAT
aniA3FA3	cctggagggaataatgacccTCCTCGGCGCGGCACTC
aniA-3R	TGACGGTTCGGGCATTCCT
aphA3-SmF	GGGTGACTAACTAGGAGGAA
aphA3-SmR	GGGTCATTATTCCCTCCAGG
gpxA-3R	GCGTTTGAAGGCTACCCCACAC
aniA-3R2	GGAAGGCGGCATAGTTGGACGA
PnorB-lacR-Bm	gacgggatcCTTGTACTGTCCCATTTTGAGAGCTCCTTTT
aniA5RA3	ttcctcctagttagtcacccCGTTTCATAATGTTTTCCTTTTGTA
norB5RA3	cctggagggaataatgacccACCAGGCGGACAACTCACGA
PaniA-lacR-Bm	gacgggatccCGTTTCATAATGTTTTCCTTTTGTA
fnr-5F1	CGCGCGGTCAAACTCGGG
fnr-3R	CGATTTAGGCGGCTGCCGG
fnr-5F2	AAACGGTCGGCGGTAGGGTAG
nsrR-5F2	AGTCGGGCGTGGTCTTTCTC
nsrR-3R	ACCACCTGACCCTGATGTGC
nsrR-5F1	CAGGGTTGTGGCAAAAGCGGT
narQ-5F1	TCCCCGCTCCCAAATAGACAAT
narP-3R	AGGGGCAAACGGAAAACACTAAAC
lacZrev	ACGACGACAGTATCGGCCTCAGG
pro-L2	AATGATGAGGGCGAAAGATTAT
aniA-lacR2	GGGATCCCGTTTCATAATGTTTTCCTTTTGT
norB-lacR2	GGGATCCCGTCCCATTTTGAGAGCTCCTTTT
R-gc-216	AATgAATAGGCTGATTTGCTAGGGG
F-gc-216	CCTAGCAAATCAGCCTATTcATT
R-gc-264	ATGAACTTAAAATGAcgCTTCTAATACTTTATA
F-gc-264	TATAAAGTATTAGAAGcgTCATTTTAAGTTCAT
F-nm-214	CCCTTAAGCAAATCAGCCTATTTATT
R-nm-214	AATAAATAGGCTGATTTGCTTAAGGGC
F-gm-143	AACAAATACATATATGATAATAACTATCATTATTC
R-gm-143	AGAATAATGATAGTTATTATCATATATGTATTTGTT
R-202nm	ATTTGCtTAaGGGCTtCGGCAaAtCAG
F-202nm	GCCTGaTtTGCCGaAGCCCtTAaGCAA

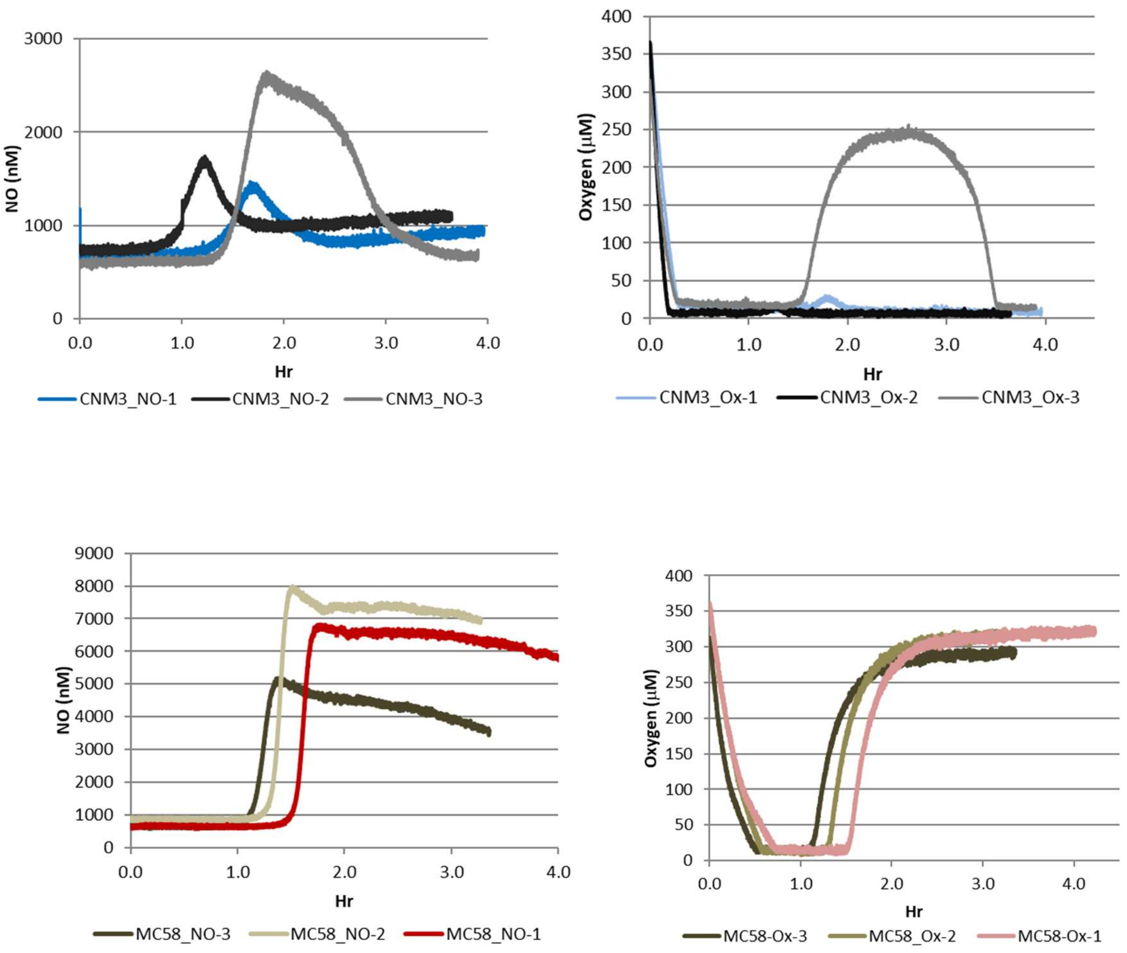

Figure S1. NO concentrations and Oxygen tension in cultures supplemented with 5 mM nitrite as measured in Clark electrode chamber. Three independent measurements for CNM3 and MC58 are plotted and the data shown in Figure 4 are colored correspondingly.

Table S3. The concentrations of NO and O_2 at the maximal levels and at 3-hr time points

	**NO (nM)**		**O_2 (μM)**	
	Maximal	at 3 hr	Maximal	at 3 hr
**CNM3-#1**	1470.88	870.51	30.55	10.18
**CNM3-#2**	1744.38	1083.99	20.37	7.87
**CNM3-#3**	2651.57	1050.66	258.27	221.71
**MC58-#1**	6820.61	6460.41	328.16	316.59
**MC58-#2**	7987.91	7200.84	322.61	315.20
**MC58-#3**	5179.62	3999.02	302.70	288.82
**T TEST***	0.006	0.007	0.051	0.034

* Two-tailed unpaired student's t test. Red: $p < 0.05$.
